# Supplementary material for: Resonant cavity phosphor
Source: Nat Commun. 2023 Oct 20;14:6661. doi: 10.1038/s41467-023-42296-1 (PMC10589315; doi:10.1038/s41467-023-42296-1)
Supplement: Supplementary file 1 — Supplementary Information [file 41467_2023_42296_MOESM1_ESM.pdf]

[Supplementary Information]

Resonant cavity phosphor

TAE-YUN LEE[1,2], YEONSANG PARK[*3,4], AND HEONSU JEON[**1,2,5]

[1]Department of Physics and Astronomy, Seoul National University, Seoul 08826, Republic of Korea

[2]Inter-university Semiconductor Research Centre, Seoul National University, Seoul 08826, Republic of Korea

[3]Department of Physics, Chungnam National University, Daejeon 34134, Republic of Korea

[4]Institute of Quantum Systems, Chungnam National University, Daejeon 34134, Republic of Korea

[5]Institute of Applied Physics, Seoul National University, Seoul 08826, Republic of Korea

*e-mail: yeonsang.park@cnu.ac.kr; **email: hsjeon@snu.ac.kr

List of Contents

S1. Calculated T, R, and A spectra of the RC-PSP 3

S2. Complex refractive indices of the CQD films 4

S3. How to determine the DBR layer numbers in the RC-PSP 5

S4. Measured T, R, and A spectra of the Ref-, RC-, and αRC-PSPs 6

S5. CQD fluorescence images taken from the Ref-, RC-, and αRC-PSPs 7

S6. PLE data measured for the red and green Ta_2O_5/SiO_2 RC-PSPs 8

S1. Calculated T, R, and A spectra of the RC-PSP

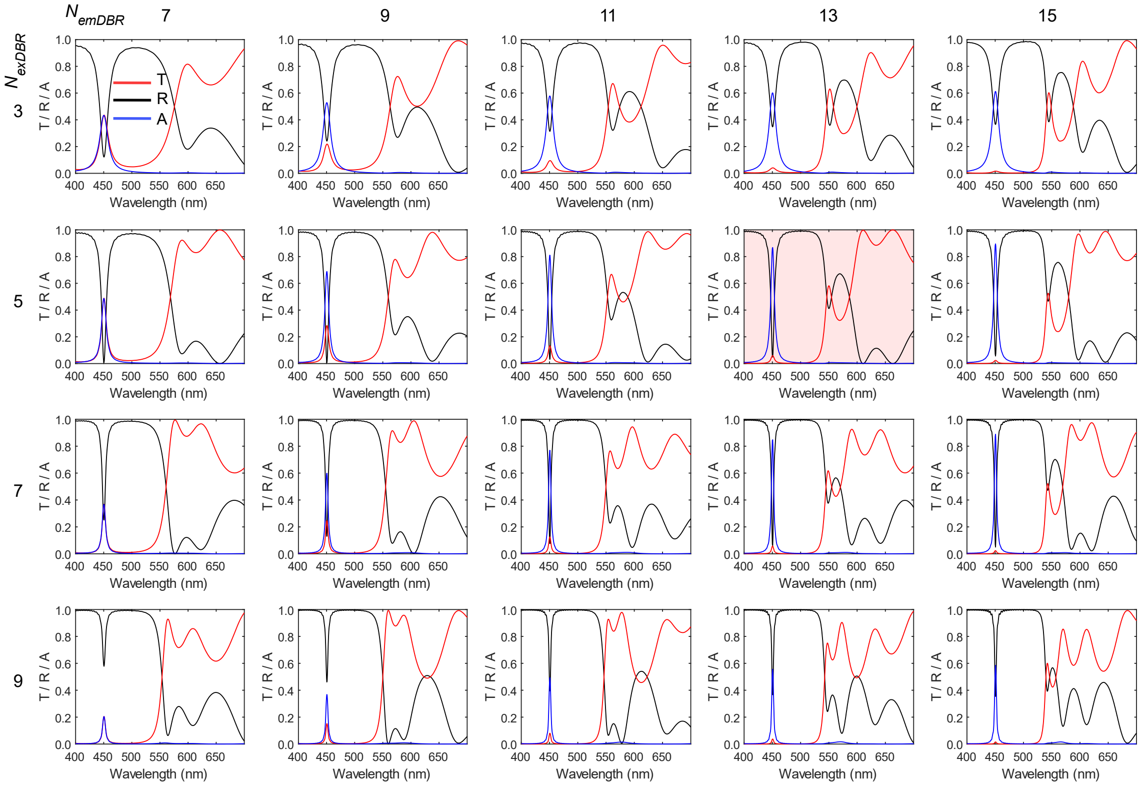

Fig. S1 | Transmittance, reflectance, and absorbance spectra calculated for the RC-PSPs composed of the TiO_2/SiO_2 DBRs. Calculation results are arranged in the matrix format of (N_{em}, N_{ex}) for N_{em} = 7, 9, 11, 13, and 15 and N_{ex} = 3, 5, 7, and 9, where N_{em} and N_{ex} are the layer numbers of the emDBR and exDBR, respectively. The RC resonance is well preserved while its peak strength and width are varied, depending on the values of N_{em} and N_{ex}. The box shaded in red highlights the RC-PSP structure chosen in the present study: (N_{em}, N_{ex}) = (13, 5).

S2. Complex refractive indices of the CQD films

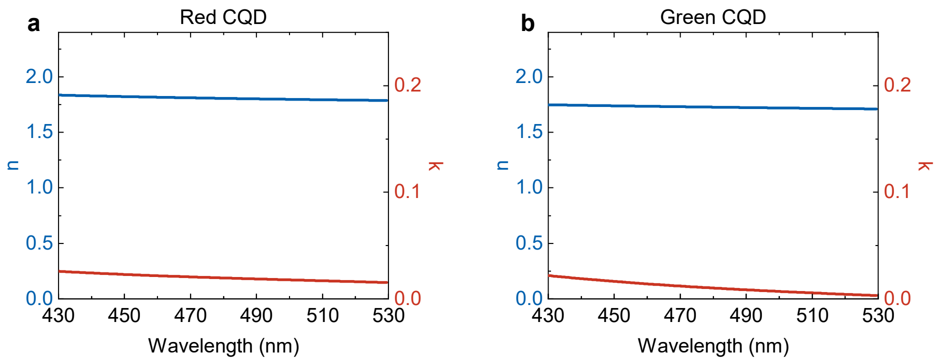

Fig. S2 | Dispersion relations of *n* and *k* of the CQD films used in the experiments. *n* and *k* measured as functions of wavelength by spectroscopic ellipsometry technique are presented for **a,** the red CQD film and **b,** the green CQD film.

S3. How to determine the DBR layer numbers in the RC-PSP

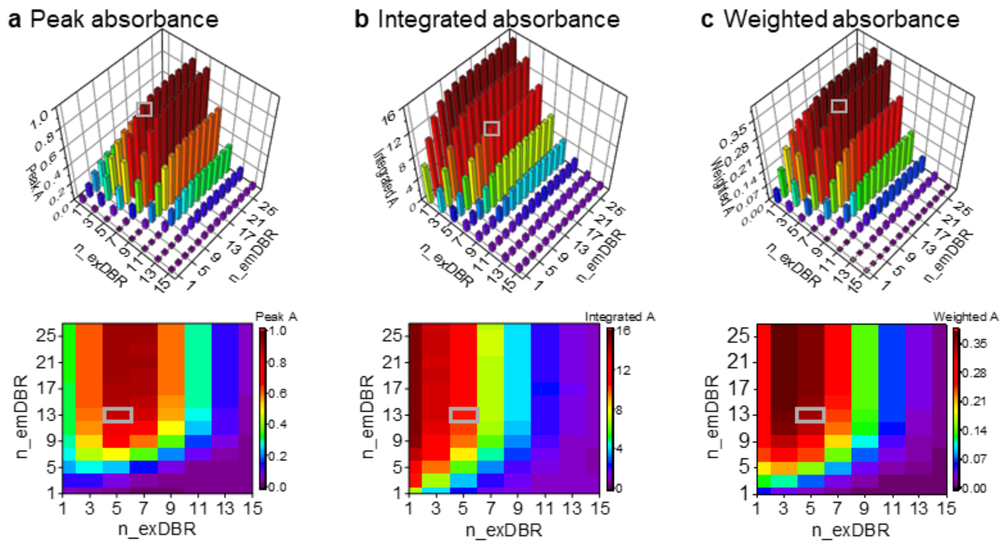

Fig. S3 | Absorbance characteristics of the RC-PSPs. Calculated absorbance spectra in Fig. S1 are analyzed to obtain **a,** the peak absorbance, **b,** integrated absorbance, and **c,** weighted absorbance. While the peak absorbance stands for the maximum at resonance, $\boldsymbol{A_{max}(\lambda_0)}$, the integrated absorbance and the weighted absorbance are defined as $\boldsymbol{\int A(\lambda) d\lambda}$ and $\boldsymbol{\int A(\lambda) I_{LED}(\lambda) d\lambda}$, respectively, where $\boldsymbol{I_{LED}(\lambda)}$ represents the emission intensity profile of a typical GaN-based blue LED, which is assumed to be a Gaussian function with center wavelength λ_0 = 450 nm and linewidth $\Delta\lambda_0$ = 20 nm. Note that the peak, integrated, and weighted absorbances represent the figure-of-merits appropriate for laser-like, broad-emission-bandwidth, and LED-like excitation sources, respectively. The results are displayed in both bar graphs (top panels) and 2D contour plots (bottom panels). The RC configuration chosen in this study, (N_{em}, N_{ex}) = (13, 5), is indicated by the rectangular boxes.

S4. Measured T, R, and A spectra of the Ref-, RC-, and αRC-PSPs

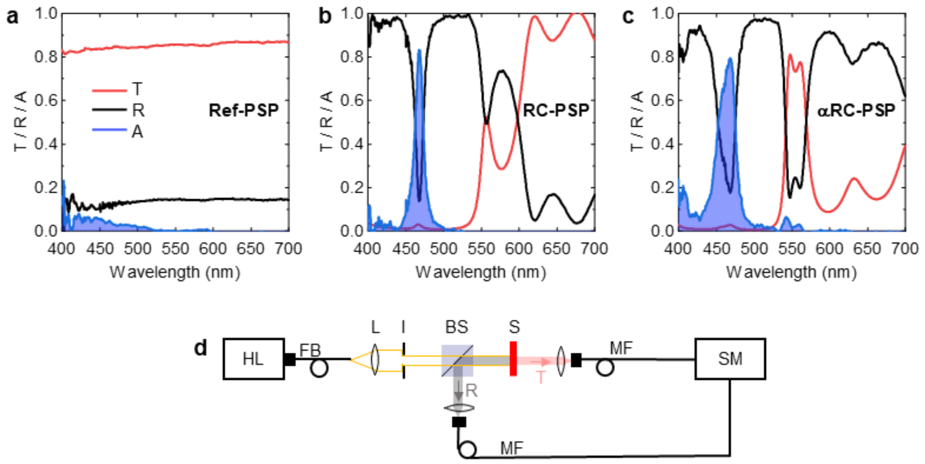

Fig. S4 | Transmittance, reflectance, and absorbance spectra measured from the fabricated phosphors. a, Ref-PSP, **b,** RC-PSP, and **c,** αRC-PSP. **d,** Schematic of the experimental setup used for transmittance and reflectance measurements (HL: halogen lamp, BS: beam splitter, SM: spectrometer, I: iris, L: lens, FB: fiber bundle, MF: multimode fiber, S: sample). While the transmittance and reflectance spectra in **a**–**c** are directly measured using the setup depicted in **d**, the absorbance spectra are deduced from the energy conservation relation: $\boldsymbol{A = 1 - T - R}$.

S5. CQD fluorescence images taken from the Ref-, RC-, and αRC-PSPs

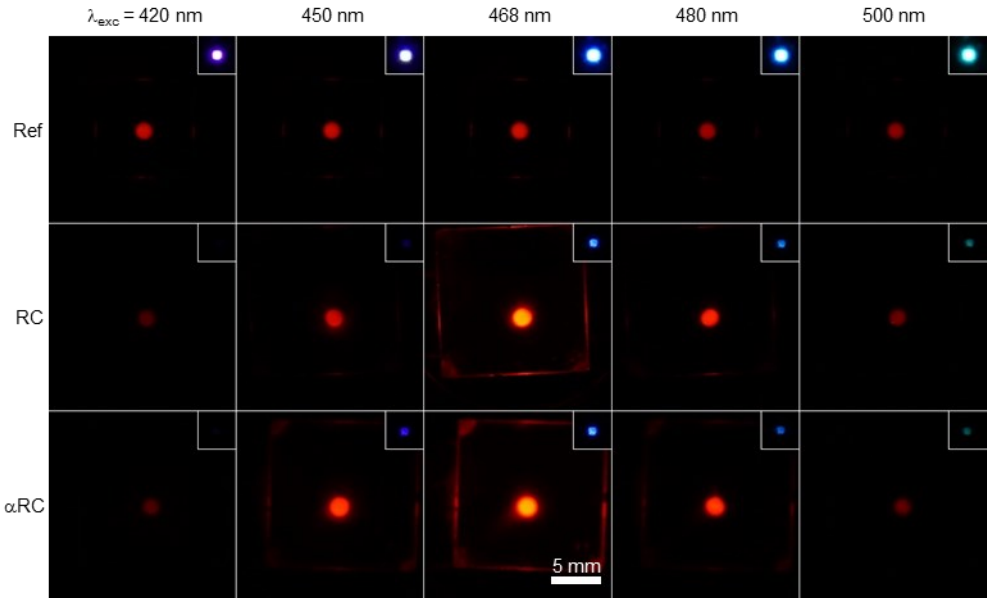

Fig. S5 | Photographs of the Ref-PSP, RC-PSP, and αRC-PSP excited at various wavelengths. CQD fluorescence images are captured through the quartz substrate for the Ref-PSP (top row), RC-PSP (middle row), and αRC-PSP (bottom row), while excited at different wavelengths near resonance: from left to right, λ_{ex} = 420 nm, 450 nm, 468 nm, 480 nm, and 500 nm. The common resonance wavelength for the both RC-PSP and αRC-PSP is λ_0 = 468 nm. A long-pass filter with cut-off wavelength 575 nm is inserted to capture the CQD fluorescence only. On the contrary, the insets are taken with a short-pass filter with cut-off wavelength 550 nm to compare the degrees of the blue leakage through the Ref-PSP, RC-PSP, and αRC-PSP. Note that the CQD fluorescence from the RC-PSP and αRC-PSP is so intense that even the CQD fluorescence guided and scattered off at the quartz substrate edges is clearly visible.

S6. PLE data measured for the red and green Ta_2O_5/SiO_2 RC-PSPs

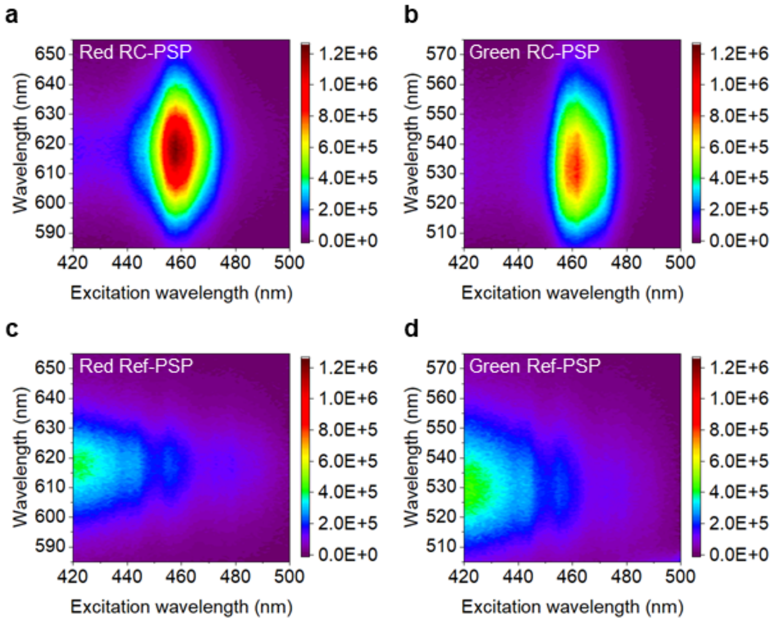

Fig. S6 | Photoluminescence excitation spectra measured from the RC-PSPs consisting of Ta_2O_5/SiO_2 DBRs. Three-dimensional contour plots of the PLE data measured for **a,** the red RC-PSP, **b,** green RC-PSP, **c,** red Ref-PSP, and **d,** green Ref-PSP. Throughout the measurements, the excitation linewidth was kept at $\delta\lambda_{ex}$ = 20 nm. The gradual increase in the PLE intensity at the short excitation wavelengths has nothing to do with the phosphor structure, but is a purely material-dependent dispersion effect: the extinction coefficient of the CQD phosphor material increases as the excitation wavelength decreases.
