# Supplementary material for: Resonant cavity phosphor
Source: Nat Commun. 2023 Oct 20;14:6661. doi: 10.1038/s41467-023-42296-1 (PMC10589315; doi:10.1038/s41467-023-42296-1)
Supplement: Supplementary file 3 — Description of Additional Supplementary Files [file 41467_2023_42296_MOESM3_ESM.pdf]

Description of Additional Supplementary Files

File: Supplementary Code

Description: MATLAB code used in the generation of figures.

res.mat: T, R, A data files related to Supplementary information S1, S3

S1.m: MATLAB source code - Supplementary information S1

S3.m: MATLAB source code - Supplementary information S3
